# Over Winter Microbial Processes in a Svalbard Snow Pack: An Experimental Approach

**DOI:** 10.3389/fmicb.2020.01029

**Published:** 2020-05-29

**Authors:** Alexandra T. Holland, Benoît Bergk Pinto, Rose Layton, Christopher J. Williamson, Alexandre M. Anesio, Timothy M. Vogel, Catherine Larose, Martyn Tranter

**Affiliations:** ^1^Bristol Glaciology Centre, School of Geographical Sciences, University of Bristol, Bristol, United Kingdom; ^2^Environmental Microbial Genomics, CNRS, École Centrale de Lyon, Université de Lyon, Lyon, France; ^3^ENOVEO, Lyon, France; ^4^Department of Environmental Science, Aarhus University, Copenhagen, Denmark

**Keywords:** snow pack, polar winter, particulate phosphorus, heterotrophic bacteria, nutrient addition

## Abstract

Snow packs cover large expanses of Earth’s land surface, making them integral components of the cryosphere in terms of past climate and atmospheric proxies, surface albedo regulators, insulators for other Arctic environments and habitats for diverse microbial communities such as algae, bacteria and fungi. Yet, most of our current understanding of snow pack environments, specifically microbial activity and community interaction, is limited to the main microbial growing season during spring ablation. At present, little is known about microbial activity and its influence on nutrient cycling during the subfreezing temperatures and 24-h darkness of the polar winter. Here, we examined microbial dynamics in a simulated cold (−5°C), dark snow pack to determine polar winter season microbial activity and its dependence on critical nutrients. Snow collected from Ny-Ålesund, Svalbard was incubated in the dark over a 5-week period with four different nutrient additions, including glacial mineral particles, dissolved inorganic nitrogen (DIN), dissolved inorganic phosphorus (DIP) and a combined treatment of DIN plus DIP. Data indicate a consumption of dissolved inorganic nutrients, particularly DIN, by heterotrophic communities, suggesting a potential nitrogen limitation, contradictory to phosphorus limitations found in most aquatic environments. 16S amplicon sequencing also reveal a clear difference in microbial community composition in the particulate mineral treatment compared to dissolved nutrient treatments and controls, suggesting that certain species of heterotrophs living within the snow pack are more likely to associate with particulates. Particulate phosphorus analyses indicate a potential ability of heterotrophic communities to access particulate sources of phosphorous, possibly explaining the lack of phosphorus limitation. These findings have importance for understanding microbial activity during the polar winter season and its potential influences on the abundance and bioavailability of nutrients released to surface ice and downstream environments during the ablation season.

## Introduction

The cryosphere, at present, covers 10% of the Earth’s surface (Anesio et al., 2009; Lutz et al., 2017) with seasonal snow packs comprising a large portion of the frozen water landscape that encompass the cryosphere, covering over one third (∼47 × 10^6^ km^2^) of the Earth’s land surface (Hinkler et al., 2008). Snow packs play many key roles as climate regulators, such as insulating soil, permafrost and supraglacial environments from sub-freezing temperatures, as well as influencing global energy and moisture budgets (Hinkler et al., 2008). Snow packs also serve as geochemical reservoirs in Arctic environments. During winter they accumulate nutrients, then release them during the spring melt, fertilizing soils, supraglacial environments, and downstream ecosystems (Kuhn, 2001; Telling et al., 2012; Larose et al., 2013b; Williamson et al., 2018). For example, Telling et al. (2012) found that on the Greenland Ice Sheet (GrIS) the prevalence of microbial nitrogen fixation in supraglacial environments related to the time since snow line retreat, suggesting that the snow pack is a main source of nitrogen to these environments.

Snow is formed in the atmosphere, which leads to scavenging of nutrients and particulates, such as ammonium, nitrate aerosols, dust and bacteria, that are used in the initiation and formation of snow crystal (Jones, 2001; Kuhn, 2001; Christner et al., 2008). The content of the resulting snow pack is thus, heavily dependent on atmospheric conditions and concentrations. Snow cover undergoes physical metamorphism during the winter season, promoted by freeze-thaw cycles and temperature gradients, which further effects the distribution of solutes and nutrients within the snow pack and ice crystals themselves (Colbeck, 1991; Kuhn, 2001; Larose et al., 2010b). Recently, microbial activity has also been found to influence snow pack nutrient species, concentration, distribution and bioavailability, and even effect overlying atmospheric concentrations (Kuhn, 2001; Amoroso et al., 2010; Fujii et al., 2010; Larose et al., 2013a; Bergk Pinto et al., 2019). For example, one study has found that microbial nitrogen cycling, occurring predominantly at the base of the snow pack, leads to basal snow enriched in dissolved nitrogen compared to surface samples (Larose et al., 2013a). Yet, research on the influence of microbial activity on nutrient cycling in snow pack environments is still in its infancy.

Presently, most research on Arctic seasonal snow pack environments focuses on the polar summer, when there is 24-h sunlight, above freezing temperatures and snow melt. This is considered to be the main growth season for microbial life residing in these environments, and an important period for nutrient export to downstream ecosystems via snow melt (Kuhn, 2001; Telling et al., 2012; Larose et al., 2013b). In contrast, there are very few studies that investigate the polar winter, which features 24-h darkness, sub-freezing temperatures, and extremely limited quantities of liquid water in the snow cover (Amoroso et al., 2010). As such, it is assumed that microbial life becomes inert during this season. However, this may not be the case. Several studies of ice core, glacial and sea ice and snow environments have reported active microbial communities during sub-freezing temperatures and low liquid water content (Carpenter et al., 2000; Junge et al., 2004; Miteva et al., 2007; Miteva, 2008, 2011; Maccario et al., 2019). For example, ice core studies have found certain psychrophiles, cold adapted organisms, capable of living at temperatures as low as −30°C (Langdahl and Ingvorsen, 1997; Price and Sowers, 2004), while Antarctic snow environments were found to contain metabolizing bacteria at −17°C (Carpenter et al., 2000). To date though, only one biogeochemical study of a cold Arctic snow pack environment during the polar winter has been conducted, which found that microbial oxidation of ammonium lead to the production and emission of NO, NO_2_ and gaseous nitrous acid (HONO) from the snow pack at levels high enough to alter the overlying atmospheric nitrogen concentration, even in the complete absence of light and at temperatures reaching −25°C (Amoroso et al., 2010). It is therefore highly possible that other macronutrient cycles in Arctic snow packs are also influenced by microbial communities during the polar winter.

Dissolved inorganic phosphorus (DIP) has long been considered the ultimate limiting nutrient in glacial environments, mainly because it is principally rock-derived (Stibal et al., 2008, 2009). Most of the phosphorus (hereafter, P) found in glacial environments is sediment bound (Säwström et al., 2002; Hodson et al., 2004, 2005). Sources of sediment and rock derived particles to snow pack and supraglacial environments are typically limited, comprised mostly of wind-blown debris from local terrestrial environments, atmospheric dust and melt-out of meteoric ice (Foreman et al., 2007; Wientjes et al., 2011; Bagshaw et al., 2013). Cryoconite, a material composed of nearby terrestrial rocks and dust deposited from the atmosphere that is later combined by ‘glue-like’ extracellular polymeric substances (EPS) secreted by cyanobacteria (Hodson et al., 2010b; Stibal et al., 2012; Yallop et al., 2012; Musilova et al., 2016), is readily found in supraglacial environments, covering up to 10% of glacier ablation zones in the Northern Hemisphere (Hodson et al., 2007, 2010a; Anesio et al., 2009). It exists both in cryoconite holes and as dispersed material on the ice surface following the melt or wash-out of the holes (Anesio et al., 2009; Hodson et al., 2010a; Telling et al., 2012, 2014; Yallop et al., 2012; Wadham et al., 2016). Cryoconite has the potential to act as a particulate inorganic phosphorus source to supraglacial environments, with potentially bioavailable phosphorus found in the particles in the order of 160 μg P g^–1^, which is well in surplus of the maximum microbial demand of about 2 μg P g^–1^ calculated for cryoconite microbial communities (Stibal et al., 2008). While cryoconite particles are not commonly found in snow pack environments, dust deposited from the atmosphere, that is later incorporated into cryoconite material, is commonly deposited to the snow pack via wet and dry deposition. Studies in other non-Arctic, P limited environments have found that rocks containing inclusions of P rich minerals, such as apatite and biotite, were highly colonized by local microbial communities and contained evidence of etching and bio-weathering (Rogers et al., 1998; Taunton et al., 2000a, b; Welch et al., 2002). To date, no study has investigated the ability or occurrence of Arctic microbial communities to access and utilize cryoconite particles as a source of P. We note that if microbial communities can access inorganic phosphorus from particles during the polar winter, then P may not be as limiting as previously considered and could indicate that other supraglacial microbial communities are capable of particulate inorganic phosphorus extraction too.

To this end, a laboratory experiment to examine the potential for microbial activity in a Svalbard snow pack during the polar winter was conducted. Dissolved nutrients and naturally occurring inorganic particulates were added to snow, which was then incubated in a cold (−5°C), dark environment. The aims of this experiment were twofold, first, to determine the dynamics of heterotrophic bacteria in a cold, dark snow pack environment, and second, to determine if the addition of dissolved and particulate inorganic nutrients effected heterotrophic abundance and community composition. In particular, to determine if heterotrophs can access the bioavailable inorganic phosphorus in cryoconite material. We hypothesize the addition of dissolved inorganic nutrients will stimulate heterotrophic activity in a cold, dark snow pack environment, leading to measurable changes in abundance and/or community composition. Further, we hypothesize a specialization of the heterotrophic community within the particulate inorganic nutrient addition.

## Materials and Methods

### Snow Collection

Snow samples were collected during a 2012 March field campaign in Ny-Ålesund (Svalbard, Norway, 78°56′ N, 11°52′ E). Freshly fallen snow samples were collected into 3 L sterile sampling bags using a sterilized Teflon shovel and stored at −20°C. Strict measures were taken to reduce human impact on sampling site and samples (Larose et al., 2009). Tyvex body suits and latex gloves were worn during sampling to avoid contamination, and gloves were worn during all subsequent handling of samples. Snow samples were maintained below 0°C during transport to Ecole Centrale de Lyon, where they were stored at −15°C until further utilization.

### Microcosm Set Up

The snow was disaggregated in sterile Whirl-pak^TM^ bags using a hammer in a −15°C cold room and transferred into two large polystyrene boxes lined with sterile Whirl-pak^TM^ bags where it was homogenized using a sterilized Teflon shovel.

**FIGURE 1:**
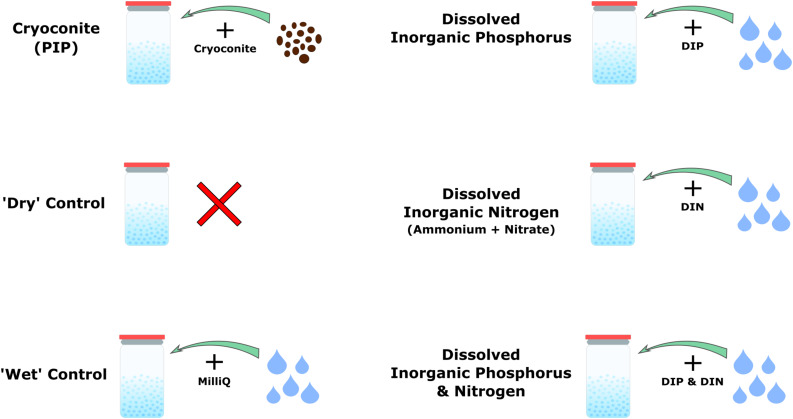
Schematic depicting the four different nutrient addition treatments and two controls.

Four nutrient addition experiments plus two controls were conducted, as shown in Figure 1. Nutrient additions consisted of particulate inorganic phosphorus (PIP), in the form of cryoconite particles described below, dissolved inorganic phosphorus (DIP), dissolved inorganic nitrogen (DIN), and DIP plus DIN. ‘Dry’ controls consisted only of snow, whereas the ‘wet’ controls consisted of snow plus 1 mL of MiliQ water to mimic the dissolved nutrient addition (Figure 1). There were five time points in total for the ‘dry’ control and the particulate phosphorus microcosms, while the ‘wet’ control, DIP, DIN, and DIP plus DIN were only sampled at the first and last time points due to experimental constraints. Each time point had four replicates per treatment. The microcosms were held in 72, 2 L glass jars, which were rinsed with 10% HCl, washed in a dishwasher using bleach and MiliQ water at 72°C and furnaced at 220°C for 20 min with foil covering the top for sterilization. 300 g of snow was weighed and placed in each sterilized jar. The respective nutrient spikes (see below) were then added and homogenized. Snow and nutrients were homogenized by mixing with a sterilized spatula for roughly 10 s each. Controls were homogenized the same as the samples. The ‘dry’ control was ‘homogenized’ in order to prevent any discrepancy between sample and control. MiliQ water blanks were also included in three time points throughout the experiment by using MiliQ in place of snow to check for nutrient leaching from the incubation bottles. 300 mg of cryoconite particles, collected from the Greenland Ice Sheet in the summer of 2017 were added for the PIP addition. The particles were furnaced at 550°C for 4 h prior to addition in order to prevent any microbial or organic carbon contamination. A ratio of 2.2 mg Phosphorus/1 g Cryoconite was assumed to determine the concentration of inorganic phosphorus added to the microcosms by the cryoconite particles (Stibal et al., 2008). 1 ml of a concentrated solution was pipetted into the snow for the DIP, DIN, and DIP plus DIN addition. DIP concentrations were chosen in order to match the concentration of inorganic phosphorus added by the cryoconite particles. A 660 ppm solution comprised of ${\text{PO}}_{4}^{3-}$- P was used to obtain a final concentration of 2.2 ppm after a 300-time snow dilution. DIN was comprised of ammonium (${\text{NH}}_{4}^{+}$) and nitrate (${\text{NO}}_{3}^{-}$), as nitrite (${\text{NO}}_{2}^{-}$) is typically below detection limits for Arctic environments (Telling et al., 2011, 2012; Wadham et al., 2016). DIN concentrations were determined from the PIP and DIP concentrations using a C:N:P ratio of 106:16:1 (Redfield et al., 1963). A 10,560 ppm solution comprised of 5,280 ppm dissolved ${\text{NH}}_{4}^{+}$ - N and 5,280 ppm dissolved ${\text{NO}}_{3}^{-}$ - N was used to obtain a final concentration of 35.2 ppm after a 300-time snow dilution. 1 ml of crystal violet stain was added to an extra microcosm in order to test our method of homogenizing dissolved nutrients into the snow. The furnaced foil was replaced on top of the jars and a lid was added to keep the foil in place. The jars were stored in the dark at −5°C until sampling.

### Sampling

The ‘dry’ control and cryoconite addition microcosms were destructively sampled every 5 days over a total of 3 weeks, by leaving the jars to melt overnight at room temperature. The ‘wet’ control and dissolved nutrient addition microcosms were destructively sampled in the same way, on the initial (T0, June 26) and final (T4, July 16) time points only. pH measurements were taken of the snow meltwater using a Consort C532 meter with an epoxy gel 1M BNC electrode (Fisher Scientific) and AVS TITRINORM^®^ pH 7 (20°C) buffer solution (VWR Chemicals). Cryoconite treatments and ‘dry’ controls were filtered using a sterile plastic syringe and a 25 mm, 0.2 μm cellulose nitrate filter (Whatman^TM^). The filtrate was collected into pre-cleaned high-density polyethylene plastic bottle (Nalgene^TM^; 30 mL) and stored at −20°C, for DIP, dissolved organic phosphorus (DOP), DIN and dissolved organic nitrogen (DON) analysis. Filters retaining the cryoconite particles were stored in sterile polypropylene tubes at −20°C prior to particulate phosphate extractions. Roughly 250 ml of meltwater from the DIP, DIN, DIN plus DIP and ‘wet’ control treatments were filtered onto sterile 47 mm, 0.2 μm Isopore^TM^ membrane Millex filters using a sterile filtration unit (Nalge Nunc International Corporation). The filters were collected into sterile polypropylene tubes and immediately stored at −20°C prior to qPCR analysis and 16S rRNA gene Illumina sequencing. The remaining meltwater was extracted using a sterile plastic syringe and filtered through a 25 mm, 0.22 μm cellulose nitrate inline syringe filter (Whatman^TM^) for DIP, DOP, DIN, and DON analysis. The filtrate was stored in a pre-cleaned high-density polyethylene plastic bottle (Nalgene^TM^; 30 mL) at −20°C. Samples were maintained at these temperatures during transport and storage at the LowTex Laboratory at the University of Bristol. Dissolved nutrient samples were thawed immediately prior to analysis using a hot water bath set at a temperature of ∼40°C. Procedural blanks were collected (*n* = 6) during the experiment, by processing deionized water in place of sample.

### Analytical Methods

#### Dissolved Nutrients

DIP (principally${\text{PO}}_{4}^{3-}$) and total dissolved phosphorus (TDP) were determined on a Gallery Plus Automated Photometric Analyzer (Thermo Fisher Scientific, United Kingdom). TDP is the sum of DIP and DOP, and was determined by digesting the samples with a sulfuric acid persulfate digestion reagent and autoclaving at 121°C for 30 min (Jeffries et al., 1979). DOP was then calculated by subtracting DIP from TDP (i.e., DOP = TDP – DIP). The limit of detection (LoD) was 4.0 ppb (${\text{PO}}_{4}^{3-}$ and TDP/DOP). LoD was determined by the mean concentration plus three times the standard deviation of procedural blanks (*n* = 6). Precision was ± 0.43% (${\text{PO}}_{4}^{3-}$) and ± 0.51% (TDP/DOP), and accuracy was + 0.12 (${\text{PO}}_{4}^{3-}$) and −7.0% (TDP/DOP), as determined from comparison with gravimetrically diluted 1000 mg L^–1^ ${\text{PO}}_{4}^{3-}$ - P certified stock standards to a concentration of 100 ppb (Sigma TraceCERT^®^). All DIP and DOP concentrations were field blank corrected, using values of 6.5 ppb, *n* = 5 and 10.9 ppb, *n* = 4 respectively.

Total dissolved inorganic nitrogen (TDIN) species include ${\text{NO}}_{2}^{-}$, ${\text{NO}}_{3}^{-}$ and ${\text{NH}}_{4}^{+}$, which were quantified as follows. ${\text{NO}}_{2}^{-}$, total oxidized nitrogen (TON) (${\text{NO}}_{2}^{-}$ + ${\text{NO}}_{3}^{-}$) and ${\text{NH}}_{4}^{+}$ were quantified spectrophotometrically using a Gallery Plus Automated Photometric Analyzer (Thermo Fisher Scientific, United Kingdom). This combination of analyses allows the original ${\text{NO}}_{3}^{-}$ concentration to be determined by subtracting ${\text{NO}}_{2}^{-}$ from TON (i.e., ${\text{NO}}_{3}^{-}$ = TON –${\text{NO}}_{2}^{-}$). Total dissolved nitrogen (TDN) is the sum of TDIN and DON and was determined by digesting the samples with a potassium persulfate, sodium hydroxide and boric acid reagent and autoclaving at 121°C for 30 min (Grasshoff et al., 1999). This process causes the oxidation of organic nitrogen compounds, which can then be measured as TON as above. DON was then calculated by subtracting the sum of the TDIN from the TDN concentration of the digested samples (i.e., DON = TDN – TDIN). LoD was determined by the mean concentration plus three times the standard deviation of procedural blanks (*n* = 5). The LoDs were 1.5 ppb (${\text{NO}}_{2}^{-}$), 5.5 ppb (TON), 14.0 ppb (${\text{NH}}_{4}^{+}$) and 5.5 ppb (TDN/DON). Precision was ± 0.19% (${\text{NO}}_{2}^{-}$), ± 1.1% (${\text{NO}}_{3}^{-}$), ± 0.36% (${\text{NH}}_{4}^{+}$) and ± 1.8% (TDN/DON), and accuracy was + 0.53% (${\text{NO}}_{2}^{-}$), −7.6% (${\text{NO}}_{3}^{-}$), −0.31% (${\text{NH}}_{4}^{+}$) and −11.5% (TDN/DON), as determined from comparison with gravimetrically diluted 1000 mgL^–1^ ${\text{NO}}_{2}^{-}$ - N, ${\text{NO}}_{3}^{-}$ - N and ${\text{NH}}_{4}^{+}$ - N certified stock standards to a concentration of 30 ppb (${\text{NO}}_{2}^{-}$) and 100 ppb (${\text{NO}}_{3}^{-}$, ${\text{NH}}_{4}^{+}$, TDN/DON) (Sigma TraceCERT^®^). All TON, ${\text{NH}}_{4}^{+}$ and DON samples were field blank (*n* = 5) corrected by subtracting values of 8.1, 31.5, and 16.8 ppb respectively. ${\text{NO}}_{2}^{-}$ field blanks fell below the LoD, 1.5 ppb, *n* = 6, so no blank correction was applied.

#### Particulate Phosphorus

**FIGURE 2:**
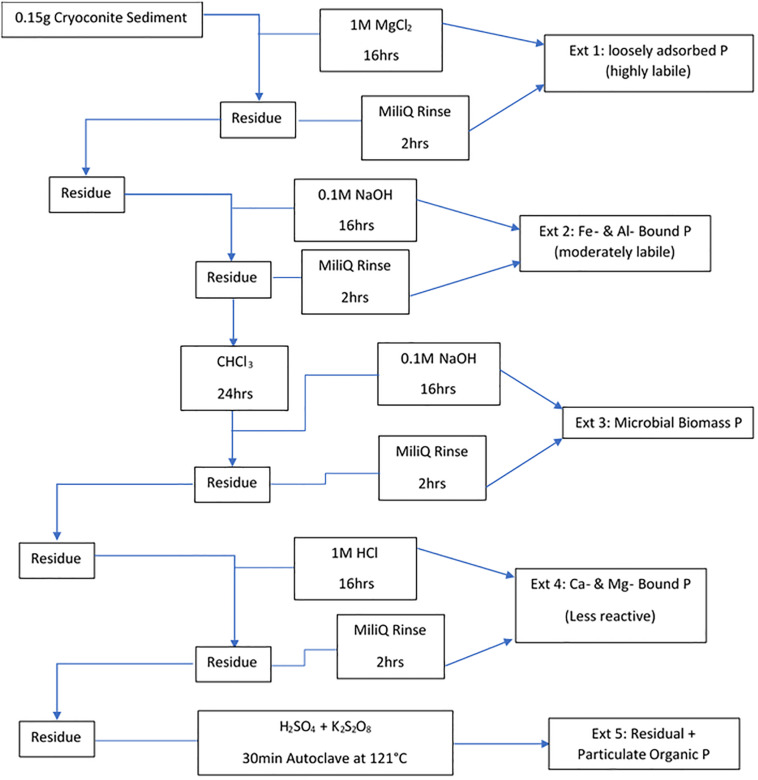
Sequential extraction method for different forms of P in the cryoconite particles added to the cryoconite treatment. All steps were done under laboratory temperature and pressure unless stated otherwise.

Filters were removed from the −20°C storage prior to extraction and left to dry in a laminar flow hood. A five-step sequential extraction scheme, adapted from Stibal et al. (2008), was then used to operationally define phase association and bioavailability of particulate P in the cryoconite used in the incubations. The scheme used by Stibal et al. (2008) comprises of loosely bound P (Ext. 1), iron and aluminum bound P (Ext. 2), calcium and magnesium bound P (Ext. 3), organic P (Ext. 4) and residual P (Ext. 5). Extraction 4, organic P, from Stibal et al. (2008) scheme was combined with Extraction 5, residual P, in our scheme as the cryoconite particles had been furnaced for organic contamination prior to addition, thus there was no need to formally quantify the organic phosphorus. Our scheme includes a step adapted from Hedley and Stewart (1982) instead, which quantifies the phosphorus incorporated into microbial biomass in the cryoconite treatment. This step most logically fits as Extraction 3 in our scheme as it uses NaOH as the solvent, the same solvent used in Extraction 2 to determine iron and aluminum bound P. A sediment: solute ratio of ∼150 mg: 3 mL used was similar to others (Hodson et al., 2004; Hawkings et al., 2016). The five-step extraction scheme used in this experiment is described in Figure 2. The content of P associated with different fractions of the cryoconite was quantified as DIP and DOP, as described above. Procedural blanks (*n* = 3) for all five extractions were conducted, using MiliQ water in place of sample to test for contamination. All DIP and DOP concentrations were blank corrected using a procedural blank. A conversion from dissolved phosphorus to particulate phosphorus concentration was applied to the cryoconite treatment and ‘dry’ control samples using the extract volume multiplied by the concentration divided by the sample weight.

#### DNA Extraction

DNA was extracted from filters of the DIP, DIN, DIN plus DIP and ‘wet’ control treatments using the DNeasy PowerWater Kit (Qiagen) following the manufacturer’s instructions. DNA was quantified using the Qubit dsDNA HS Assay Kit (Thermo Fisher Scientific).

DNA was extracted from the particles in the cryoconite addition treatment using the protocol outlined in Nicol and Prosser (2011). Briefly, 0.5 mL CTAB phosphate buffer and 0.5 mL of phenol:chloroform:isoamyl alcohol (25:24:1) was added to each sample. Following cell lysis in a bead beater (30 s, 4 m s^–1^), samples were centrifuged and the upper aqueous layer retained. Residual phenol was removed by the addition of 0.5 mL of chloroform:isoamyl alcohol, followed by centrifugation and retention of the upper aqueous layer. DNA was precipitated using 30% PEG/NaCl solution with an overnight incubation (5 degrees). The DNA pellet was collected by centrifugation, washed with 70% ethanol and resuspended in sterile molecular grade H_2_O. DNA was also extracted from untreated, furnaced cryoconite, as described above, to act as a control and test for any remaining DNA after sterilization. Two separate DNA extraction methods were utilized due to the differing material from which the DNA was extracted. DNA extracted from filters utilized a method developed for low biomass filer extractions, while a method developed for sediment extraction was better suited for the cryoconite addition treatment.

#### 16S rRNA qPCR

Real-time qPCR analyses on the 16S rRNA genes were carried out to approximate the concentration of bacterial cells per ml of melted snow or gram of cryoconite knowing that bacteria might have more than one copy of 16S rRNA per genome. The V3 region of the 16S rRNA gene was amplified using the GoTaq qPCR Master Mix (Promega, reference A6001) using primers sequences 341F and 534R (Muyzer, 1996; Watanabe et al., 2001) on a Rotorgene 3000 machine (Qiagen). The reaction mixture of 20 μL contained 10 μL of GoTaq qPCR Master Mix, 2 μL of DNA and RNAse-free water to complete the final 20 μL volume. The qPCR 2-steps program consisted of an initial step at 95°C for 2 min for enzyme activation, then 35 cycles of 5 s at 95°C and 20 s at 60°C hybridization and elongation. A final step was added to obtain a denaturation from 55 to 95°C with increments of 1°C s^–1^. The amplicon length was around 200 bp. PCR products obtained from DNA from a pure culture of *Escherichia coli* were cloned in a plasmid (pCR2^TM^.1-TOPO^®^ vector, Invitrogen) and used as standard after quantification with the Broad-Range Qubit Fluorometric Quantification (Thermo Fisher Scientific).

#### 16S rRNA Sequencing and Bioinformatics

Microbial community structure was determined by MiSeq Illumina amplicon sequencing of the bacterial V3–V4 region of the 16S rRNA gene using the Library Preparation Workflow recommended by Illumina (Illumina, Inc., San Diego, CA, United States). The V3–V4 region of the 16S rRNA gene was amplified using the Platinum Taq Polymerase (Thermo Fisher Scientific) using the primer set 783F – 1046R from Klindworth et al. (2013) resulting in the following sequences: 5′-TCGTCGGCAGCGTCAGATGTGTATAAGAGACAGCCTA CGGGNGGCWGCAG-3′ as the forward primer sequence, and 5′-TCGTCGGCAGCGTCAGATGTGTATAAGAGACAGC CTACGGGNGGCWGCAG-3′ as the reverse primer sequence (Illumina, Inc., San Diego, CA, United States). The PCR program used was: 95°C for 3 min, 35 cycles of 95°C for 30 s, 55°C for 30 s and 72°C for 30 s, then a final step of 72°C for 5 min. Paired end sequencing was then carried out on a MiSeq sequencer (Illumina) at the laboratory in Lyon. Sequencing primers were removed using CutAdapt and filtered, trimmed and processed using Dada2. Taxonomy was assigned to the inferred sequence variants using the Dada2 formatted RDP dataset (RDP trainset 16).

### Data Analysis

All geochemical measurements below the LoD were considered to be 0 for all statistical analyses. All DIN, DON, DIP, and DOP data were water blank-corrected using values from the respective procedural blanks. All PIP and POP data were water blank-corrected using values from the respective procedural blanks. Additionally, all blank corrected values that were negative were assumed to be 0 for all statistical analyses. Dada2 results and annotations were imported into R (R Development Core Team, 2011) and analyzed with the R package ‘phyloseq’ (McMurdie and Holmes, 2013). Amplicon sequence variants (ASVs) not taxonomically assigned to Bacteria were excluded from further analysis. Samples were rarefied to equal the sample with the lowest read counts using the ‘rarefy even depth’ phyloseq function prior to Alpha diversity calculations with the ‘estimate richness’ function. ASV count matrices were normalized by relative abundance prior to statistical analyses. Statistical analyses were performed in RStudio v.1.1.414 (RStudio Team, 2018). Identification of statistical differences between nutrient concentrations, bacterial abundance of dissolved nutrient treatments, sample date and treatment type were achieved using two-way and one-way analysis of variance (ANOVA) comparisons, with *post hoc* Tukey HSD analysis applied to all significant ANOVA results. *t*-Tests were used to compare observed Alpha diversity measurements for each treatment between time points. Homogeneity of variance and normality of distribution were tested prior to all parametric analyses, and model assumptions were verified by examination of model criticism plots.

## Results

### Dissolved Inorganic Nutrients

**FIGURE 3:**
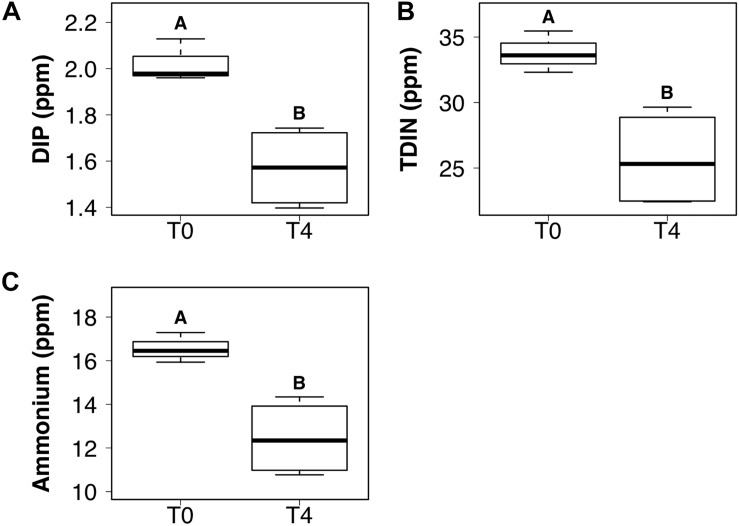
Boxplots showing the median, upper and lower interquartile range of dissolved inorganic phosphorus (DIP) **(A)**, total dissolved inorganic nitrogen (TDIN) **(B)** and ammonium **(C)** concentrations for the combined DIP and dissolved inorganic nitrogen (DIN) treatment at the initial (T0) and final (T4) time points. *Uppercase letters* denote homogeneous subsets derived from *post hoc* Tukey HSD analysis on a significant two-way ANOVA in relation to the time point and treatment.

**FIGURE 4:**
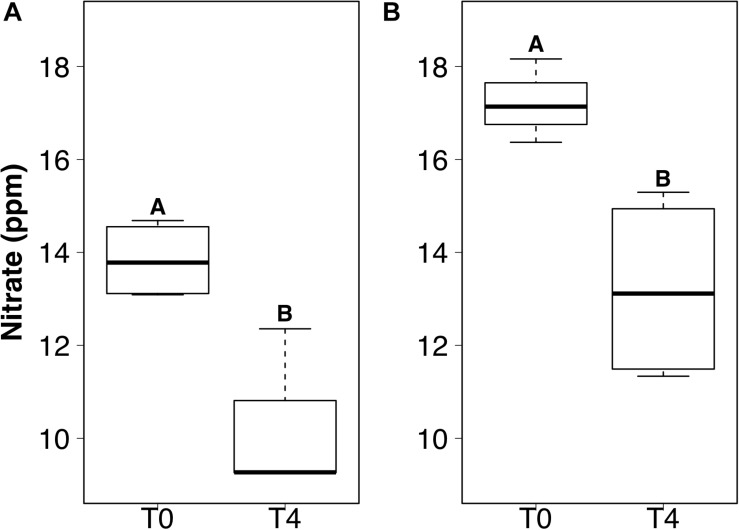
Boxplots showing the median, upper, and lower interquartile range of nitrate concentrations for DIN treatment **(A)** and combined dissolved inorganic phosphorus (DIP) and DIN treatment **(B)** at the initial (T0) and final (T4) time points. *Uppercase letters* denote homogeneous subsets derived from *post hoc* Tukey HSD analysis on a significant two-way ANOVA in relation to the time point and treatment.

DIP and TDIN decreased significantly over the 5-week sampling period. The decrease in DIP and TDIN concentrations was not homogenous across all treatments, however. Both DIP and TDIN concentrations decreased significantly in the combined DIN plus DIP treatment only (*F*_1_,_10_ = 17.5, *p* < 0.01 and *F*_1_,_10_ = 1.0, *p* < 0.01, respectively) (Figures 3A,B). DIP concentrations in the combined treatment decreased from 2.02 ± 0.05 to 1.57 ± 0.09 ppm while TDIN concentrations decreased from 33.8 ± 0.92 to 25.7 ± 1.9 ppm. TDIN is composed of ${\text{NH}}_{4}^{+}$ and ${\text{NO}}_{3}^{-}$, as previously described, therefore, changes in each species of nitrogen were investigated in order to determine which N species was in higher demand. ${\text{NH}}_{4}^{+}$ decreased significantly from 16.6 ± 0.39 to 12.4 ± 0.87 ppm in the combined treatment only (*F*_1_,_10_ = 1.6, *p* < 0.05) (Figure 3C). ${\text{NO}}_{3}^{-}$ was the only added dissolved nutrient that significantly decreased in both the single and combined treatments (*F*_1_,_10_ = 0.1, *p* < 0.05, for both) (Figure 4). ${\text{NO}}_{3}^{-}$ concentrations decreased from 13.8 ± 0.42 to 10.3 ± 1 ppm in the DIN addition treatment and from 17.2 ± 0.52 to 13.2 ± 1 ppm in the combined treatment. No significant change occurred in any dissolved nutrient concentration in the ‘wet’ control, nor in DIP concentrations in the DIP treatment.

### Bacterial Abundance and Taxonomy

**FIGURE 5:**
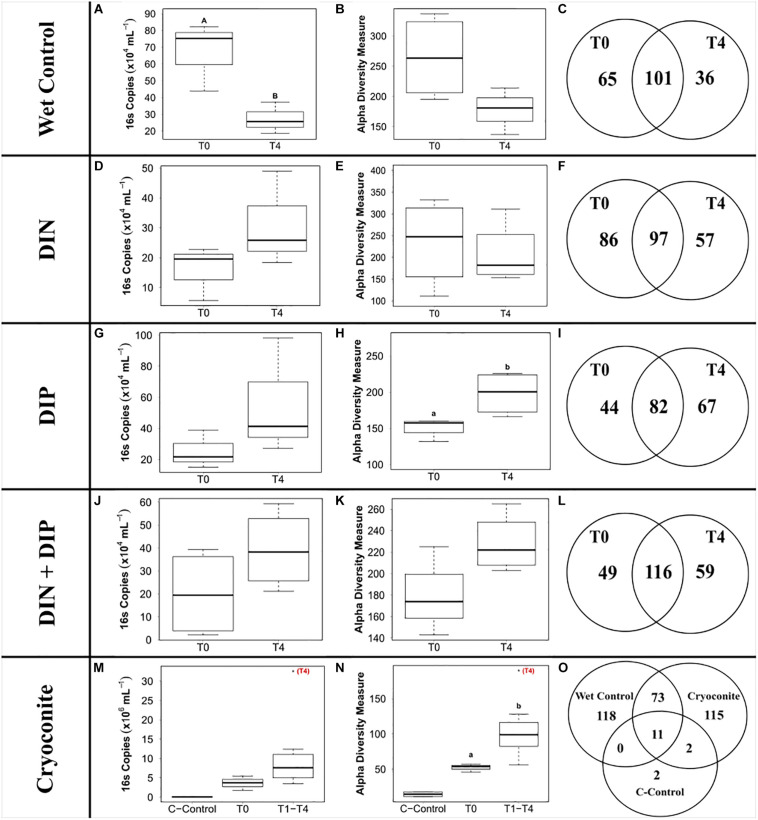
Boxplots showing the median, upper and lower interquartile range of bacterial abundance **(A,D,G,J,M)** and observed Alpha diversity measurement **(B,E,H,K,N)** and Venn diagrams depicting genera richness **(C,F,I,L,O)** for all treatments. T0 and T4 represent initial and final time point, respectively. C-Control in the cryoconite treatment represents untreated, furnaced cryoconite control. DIN and DIP represent dissolved inorganic nitrogen and dissolved inorganic phosphorus, respectively. *Uppercase letters* denote homogeneous subsets derived from *post hoc* Tukey HSD analysis on a significant two-way ANOVA in relation to treatment and time point. *Lowercase letters* denote *t*-test comparisons in relation to time point.

The ‘wet’ control bacterial abundance was the only treatment that exhibited a significant decrease in abundance between the initial and final time points (*F*_3_,_20_ = 6.27, *p* < 0.05) (Figure 5A). Bacterial abundance in all dissolved nutrient addition treatments were elevated in the final time point compared to the initial time point (Figures 5D,G,J). Bacterial abundance in the cryoconite treatment displayed an overall increasing trend across the timeseries, with all time points having at least a two order of magnitude increase compared to the untreated, furnaced cryoconite control and the final time point (T4) containing an order of magnitude higher bacterial abundance compared to all other time points (Figure 5M).

**FIGURE 6:**
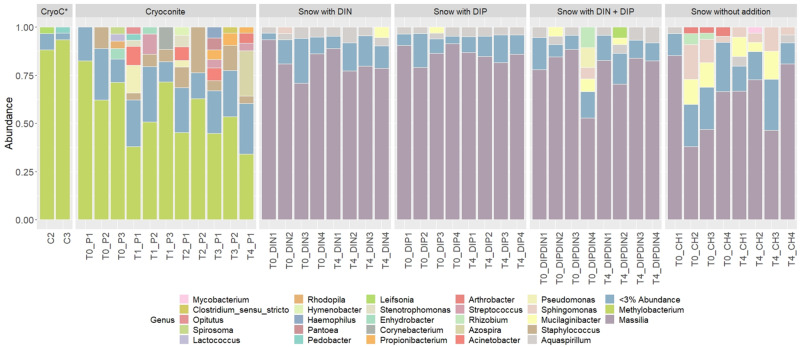
Relative abundances of taxa representing > 5% abundance and classified to genus level across all treatments and time points. CryoC* represents untreated, furnaced cryoconite control, with C2 and C3 representing the two replicates. T0–T4 represent time points 0–4, respectively. DIN and DIP represent dissolved inorganic nitrogen and dissolved inorganic phosphorus, respectively. P/DIN/DIP/DIPDIN/CH 1–4 represent the four replicates for each time point per treatment.

441 genera from 25 phyla were detected across the 4 treatments and the ‘wet’ control. Betaproteobacteria, more specifically *Massilia*, dominated the dissolved nutrient addition treatments and ‘wet’ control, whereas Alphaproteobacteria, more specifically *Methylobacterium*, had the highest abundance in the cryoconite treatment (Figure 6). There was also a clear diversification of the microbial community present in the cryoconite treatment compared to the untreated, furnaced control cryoconite particles, as only two common genera were detected, *Chryseobacterium* and *Propionibacterium* (Figures 5O, 6). Alpha diversity measurements revealed an overall decrease in genera richness between T0 and T4 for the ‘wet’ control and DIN treatment, as seen in Figures 5B,C,E,F. Alpha diversity increased over the time series for both the combined treatment (Figures 5K,L) and DIP treatment, with a significant increase found in the DIP treatment (*t* = −2.82, *p* < 0.05) (Figures 5H,I). Alpha diversity also increased significantly after T0 in the cryoconite treatment (*t* = −3.23, *p* < 0.05) (Figure 5N).

### Cryoconite Particulate Phosphorus

**FIGURE 7:**
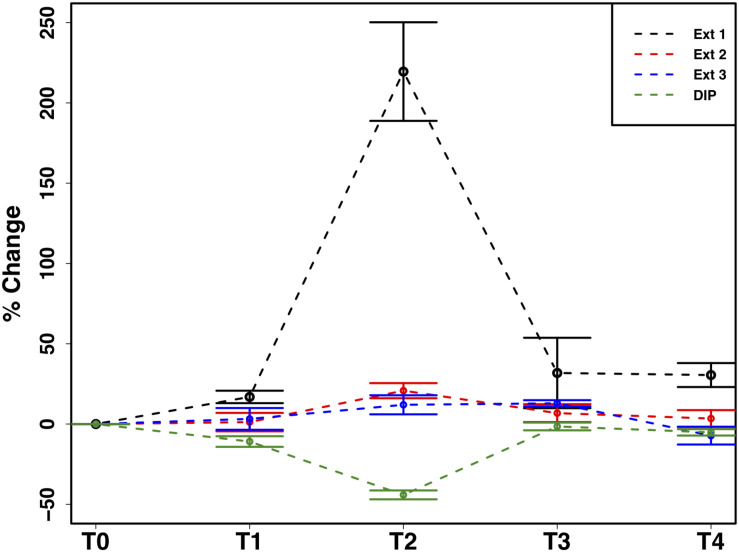
Mean ± SE of the percent change in particulate phosphorus content of cryoconite particles (Ext. 1–3) and dissolved inorganic phosphorus (DIP) concentration of filtrate from cryoconite treatment throughout the time series, *n* = *4* for each time point of each extraction.

**FIGURE 8:**
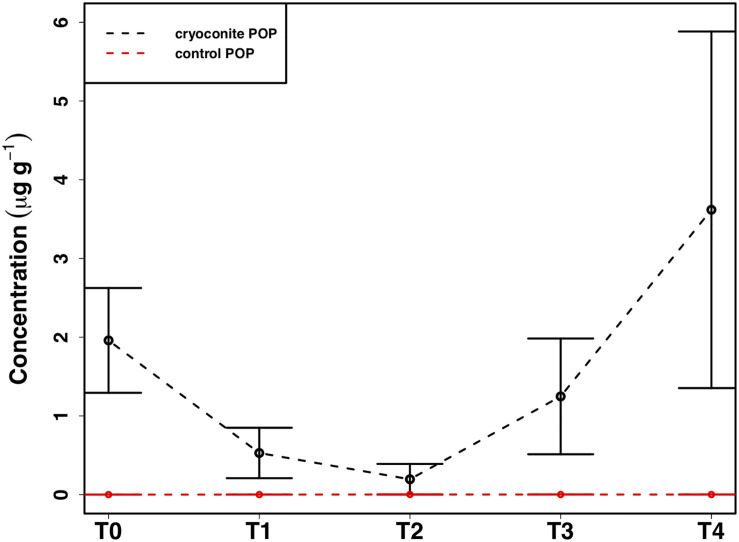
Mean ± SE of the particulate organic phosphorus (POP) content of heterotrophic biomass associated with cryoconite particles in the cryoconite treatment throughout the time series and the ‘dry’ control, *n* = *4* for each time point of each treatment.

The mean total PIP content in the cryoconite particles added to the microcosms was 649.7 ± 46.8 μg g^–1^, which falls within the range of P content within most rock types in the Earth’s crust (230–670 μg P g^–1^) (Hodson et al., 2004). Most PIP (82.8 ± 0.74%) was present in Extract 2 (‘Fe- and Al-bound’), followed by Extract 4 (‘Ca- and Mg-bound’; 8.8 ± 0.52%), Extract 5 (‘Residual + Organic P’; 4.4 ± 0.11%), Extract 3 (‘Microbial Biomass’; 4.3 ± 0.35%) and Extract 1 (‘Loosely adsorbed P’; 0.51 ± 0.23%). One-way ANOVAs revealed significant changes in PIP content in Extracts 1–3 (Ext. 1: *F*_4_,_15_ = 30.9, *p* < 0.0001, Ext. 2: *F*_4_,_15_ = 6.8, *p* < 0.001, Ext. 3: *F*_4_,_15_ = 3.5, *p* < 0.01). A spike in PIP content found in Extracts 1–3 occurring on July 6^*th*^, time point 2 (T2), midway through the time series, drives this trend. DIP concentrations quantified from the filtrate of the cryoconite treatment revealed a significant decrease in concentration on the same date (*F*_4_,_15_ = 84.3, *p* < 0.0001). This relationship can be seen in Figure 7, which depicts the percent changes for Extracts 1–3 and DIP (*F*_4_,_15_ = 27.2, *p* < 0.0001, *F*_4_,_15_ = 3.1, *p* < 0.01, *F*_4_,_15_ = 3.1, *p* < 0.01, and *F*_4_,_15_ = 58.6, *p* < 0.0001, respectively). DOP and POP results from the filtrate and extracts displayed no change over time, apart from Extract 3. Extract 3, which quantified the organic phosphorus within the microbial biomass in the cryoconite treatment, demonstrated an initial decrease, followed by a sudden increase after T2 (Figure 8).

## Discussion

In this present study we show evidence of microbial community dynamics and their effect nutrient cycling within a simulated cold, dark snow pack environment. This has implications for how we view microbial activity during the polar winter, a season that has previously been considered inert. As the 24-h of darkness and subfreezing temperatures of the polar winter can comprise up to 6 months of the year, it is important to understand microbial influences on nutrient cycling as it could affect nutrients released during the ablation season.

### Varied Heterotrophic Community Response to Dissolved Nutrient Additions

We demonstrate here the potential for active microbially-mediated nitrogen cycling in snow-packs, a dynamic already known to occur during spring and summer (Larose et al., 2013a), under simulated polar winter conditions. A clear community response was detected upon the addition of DIN, with an elevated final abundance and subsequent decline in genera richness suggesting potential specialization of heterotrophic communities, with genera better adapted to a nitrogen enriched environment potentially outcompeting other less suited bacteria (Figures 5D–F). This was supported by the presence throughout the time series of bacteria associated with the nitrogen cycle, e.g., *Bacillus* and *Caulobacter* genera known to perform N fixation (Różycki et al., 1999) and as efficient ${\text{NH}}_{4}^{+}$ scavengers (Poindexter, 1981), respectively, and the emergence of others, e.g., *Nitrospira* known for the oxidation of nitrite to nitrate (Spieck and Bock, 2015), by the final time point. As high, irregular inputs of nitrogen are common during the polar winter, via wet and dry deposition (Björkman et al., 2014), specialization in associated heterotrophic communities is likely to occur *in situ*, with consequences for the nitrogen cycle. For example, changes in bacterial assemblage composition associated with DIN addition during the present study likely underlaid the observed preferential consumption of ${\text{NO}}_{3}^{-}$ over ${\text{NH}}_{4}^{+}$, thus driving variability in the relative abundance of nitrogen species through time (Figure 4A), consistent with spring and summer *in situ* observations (Larose et al., 2013a).

In contrast to the assertion of DIP as the key limiting nutrient in glacial environments (Hodson et al., 2004; Stibal et al., 2008, 2009), DIP was only measurably consumed in the combined dissolved nutrient treatment during the present study; for which we propose two possible explanations. Firstly, it is likely that the abundance of DIN influenced the efficiency of DIP consumption, as evidenced by DIP being measurably consumed in the combined treatment only. With the absence of DIN in the DIP treatment, reduced DIP consumption would be required to maintain stoichiometric homeostasis (e.g., a C:N:P ratio of 20:4:1 for freshwater heterotrophic bacteria; Fagerbakke et al., 1996; Vrede, 1998), whereas in the combined treatment, both DIN and DIP were consumed effectively. Second, DIP may not be as limiting in supraglacial environments as previously believed (Hodson et al., 2004; Stibal et al., 2008, 2009), considering that DIN was consistently consumed during the present study when concentrations were artificially elevated. Such trends have also been reported in recent studies concerning both snow pack and surface ice environments, which suggested DIN to be in higher demand than DIP (Larose et al., 2013a; Holland et al., 2019). Overall, there is a clear response by the microbial community to all nutrient additions over the course of just 5-weeks in a cold, dark snow pack, indicating that the 24-h darkness and subfreezing temperatures of the polar winter may not be as limiting as previously thought.

### Potential Extraction of Particulate Phosphorus

In addition to the utilization of dissolved nutrient resources, we further demonstrate the potential for microbial extraction of particulate phosphorus from cryoconite particles in snow pack environments in the present study. Inverse relationships observed between DIP and PIP pools suggested the capacity for phase changes between dissolved and loosely bound phosphorous fractions. For example, a simultaneous increase in PIP and decrease in DIP concentrations at T2 (Figure 7) indicated rebinding of DIP to available receptor sites on cryoconite particles (most likely as Extract 1), producing new, loosely bound PIP accessible to microbial communities via different methods such as carbonatation weathering, dissolution or acidification through the production of organic acids (Welch et al., 2002), as described below. DIP adsorbed onto particles, whether as loosely bound P or on poorly ordered Fe- and Al- oxyhydroxides, has been found to be readily bioavailable in cryospheric environments (Hodson et al., 2004; Stibal et al., 2008). Extract 1, the most labile and readily bioavailable form of PIP (Stibal et al., 2008), accounted for the smallest portion of total PIP extracted during the present study, suggesting consumption by microbial communities. Results from the present study are analogous to those in sediment rich, oligotrophic aquatic environments, whereby P adsorption to particulates has a significant impact on the fate and distribution of bioavailable P in the environment (Müller et al., 2006). An initial decrease in P within the microbial biomass, followed by an increase after T2, suggests a possible utilization of internal phosphorus stores, perhaps through storage in polyphosphates, before being able to access the newly adsorbed, bioavailable P (Figure 8). This storage and utilization of polyphosphate molecules as an energy source has been well-documented in many other heterotrophic communities (Kornberg, 1995; White et al., 2010; Achbergerová and Nahálka, 2011; Tocheva et al., 2013).

P liberation from particulate sources has previously been documented by microbes via etching, production of organic ligands, influencing the solution saturation state or most commonly lowering the pH either at the mineral surface or the bulk pH through the production of organic acids (Welch et al., 2002). The pH of the cryoconite treatment in the present study varied only slightly with an average pH of ∼ 6.02 ± 0.13. Even though our pH was slightly acidic, production of organic acids typically lower the bulk pH to between 3.5 and 5 (Welch et al., 2002), if microbial acidification was used to mine the loosely adsorbed P, then the pH may have only been altered at the particle surface, enough to access the labile P, but not enough to effect the bulk pH, or they utilized an alternate method such as the production of pyruvate, which has also been found to aid the dissolution of P containing minerals without altering pH (Welch et al., 2002).

### Increased Abundance and Diversity Overtime in Particulate Associated Heterotrophic Community

We demonstrate a potential colonization of the cryoconite particles over time by genera found in the snow pack environment, as suggested by a combined increase in bacterial abundance and diversity over time in the cryoconite treatment and compared to the untreated, furnaced cryoconite control (Figures 5M,N). A clear diversification in the main genera found in the cryoconite treatment compared to the ‘wet’ control and untreated, furnaced cryoconite is also present (Figure 6), indicating that certain genera from the snow pack environment might be better suited for colonizing the particles. Only 73 shared genera were detected between the treated cryoconite and ‘wet’ control, representing the genera that could act as early particulate colonizers (Figure 5O). For example, *Massilia* is found in both the treated cryoconite and ‘wet’ control. *Massilia* is not only a Betaproteobacteria, a class which has been previously identified as early colonizing bacteria with high abundance in snow pack environments that may play a key role in mineral weathering in debris covered glaciers (Pianka, 1970; Fierer et al., 2007, 2010; Larose et al., 2010a; Zumsteg et al., 2012; Franzetti et al., 2013; Hell et al., 2013), but also a genera that has species linked to solubilizing phosphate (Zheng et al., 2017). In fact, there is a clear increase in overall Betaproteobacteria abundance in the final time point of the cryoconite addition treatment compared to previous time points (Figure 6). Other non-betaproteobacteria such as *Arthrobacter*, that have been shown to assist in mineral dissolution by lowering pH, are also found among the 73 potential colonizers (Barker et al., 1998; Welch et al., 2002). Based on the notable increase in bacterial abundance and Alpha diversity after T0 (Figures 5M,N), it seems likely that given more time the colonization, and potential utilization, of the particles by microbes would have continued to increase. Any potential colonization or extraction of particulate inorganic substrates by heterotrophic communities in snow pack environments is likely to occur in other supraglacial or Arctic environments as snow packs have been considered to seed microbial communities in underlying environments during the ablation season (Musilova et al., 2015).

## Conclusion

Our study indicates the presence of an active heterotrophic community in a cold (−5°C), dark snow pack environment. Snow collected from a seasonal snow pack in Ny-Ålesund, Svalbard was incubated with dissolved and particulate nutrient additions to determine whether the additions, in particular the naturally occurring inorganic substrate, affected heterotrophic abundance or community composition. Our results suggest a specialization within the heterotrophic community of genera known to influence N cycling in the presence of excess DIN, but diversification when abundant DIP is present. As high inputs of N are common to snow pack environments during the polar winter, this specialization may occur *in situ*, leading to efficient N cycling by heterotrophic bacteria. We also find strong evidence for the utilization of particulate phosphorus sources from an inorganic glacial sediment by the heterotrophic community, which also exhibits colonization by certain genera from the snow pack environment, e.g., *Betaproteobacteria*, known to be early colonizers of particulates. The ability of heterotrophic bacteria to access particulate phosphorus in snow pack and potentially other supraglacial environments may thus be a key factor influencing survival in oligotrophic glacial environments. Overall, we demonstrate that changes in heterotrophic community abundance and composition in snow pack environments influence nutrient cycling during the polar winter, which in turn impacts the speciation, abundance and bioavailability of nutrient resources relative to their depositional state, and thus their roles in supraglacial and downstream ecosystems during the ablation season.

## Data Availability Statement

Sequencing files can be accessed at http://ftp-adn.ec-lyon.fr/Holland_2020/. The full datasets generated for this study are available upon request to the corresponding author.

## Author Contributions

AH, BB, AA, TV, CL, and MT conceived and designed the study. CL collected the snow and analyzed the 16S qPCR samples with the assistance of RL. RL extracted the DNA, sequenced the rRNA and ran bioinformatics on the samples with the assistance of BB. AH wrote the manuscript with inputs from CW, CL, RL, AA, and MT. All authors reviewed the final manuscript.

## Conflict of Interest

RL was employed by ENOVEO. The remaining authors declare that the research was conducted in the absence of any commercial or financial relationships that could be construed as a potential conflict of interest.
